# Plasmon-Enhanced Fluorescence of Single Quantum Dots Immobilized in Optically Coupled Aluminum Nanoholes

**DOI:** 10.1021/acs.jpclett.3c00468

**Published:** 2023-02-27

**Authors:** Yupeng Yang, Apurba Dev, Ilya Sychugov, Carl Hägglund, Shi-Li Zhang

**Affiliations:** † Division of Solid-State Electronics, Department of Electrical Engineering, The Ångström Laboratory, Uppsala University, SE-751 03 Uppsala, Sweden; ‡ Division of Photonics, Department of Applied Physics, School of Engineering Sciences, KTH Royal Institute of Technology, SE-100 44 Stockholm, Sweden; § Division of Solar Cell Technology, Department of Materials Science and Engineering, The Ångström Laboratory, Uppsala University, SE-751 03 Uppsala, Sweden

## Abstract

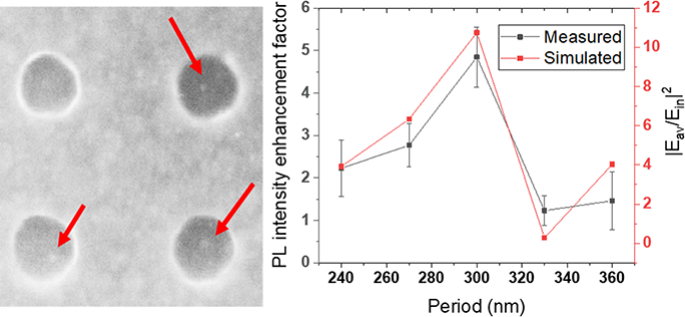

Fluorescence-based optical sensing techniques have continually been explored for single-molecule detection targeting myriad biomedical applications. Improving signal-to-noise ratio remains a prioritized effort to enable unambiguous detection at single-molecule level. Here, we report a systematic simulation-assisted optimization of plasmon-enhanced fluorescence of single quantum dots based on nanohole arrays in ultrathin aluminum films. The simulation is first calibrated by referring to the measured transmittance in nanohole arrays and subsequently used for guiding their design. With an optimized combination of nanohole diameter and depth, the variation of the square of simulated average volumetric electric field enhancement agrees excellently with that of experimental photoluminescence enhancement over a large range of nanohole periods. A maximum 5-fold photoluminescence enhancement is statistically achieved experimentally for the single quantum dots immobilized at the bottom of simulation-optimized nanoholes in comparison to those cast-deposited on bare glass substrate. Hence, boosting photoluminescence with optimized nanohole arrays holds promises for single-fluorophore-based biosensing.

Fluorescence-based optical detection techniques constitute a versatile toolbox that has been widely applied in bio-imaging,^1−3^ DNA sequencing,^4,5^ biomolecular studies,^6,7^ etc. A large fluorescence signal-to-noise ratio (SNR) is highly desired in order to attain high fidelity in sensing. There are two ways to improve SNR: amplifying the fluorescence source signal and lowering the background noise. Several alternatives exist to achieve high signal intensity. Choosing bright fluorophores is a natural consideration.^8,9^ To enhance the fluorescence intensity, various metallic nanostructures have been studied^10−12^ by exploiting their plasmonic properties with matched incident light. The fluorescence-enhancement factor can be as large as thousands.^13,14^

Quantum dots (QDs), among the brightest fluorophores, are semiconductor nanoparticles several nanometers in diameter.^15^ In comparison with other traditional fluorophores such as organic dyes or fluorescent proteins, state-of-the-art QDs are characterized by broad excitation ranges but narrow emission peaks, large Stokes shift, good stability, and long lifetime.^15^ Moreover, they can be conjugated with various biomolecules by matured surface chemistry techniques for biological applications.^16−18^ Thus, QDs have been widely applied in biology,^19,20^ energy harvesting,^21,22^ displays,^23,24^ quantum information,^25,26^ etc.

Single QD-based studies have thus far shown promising results for single-molecule detection^27,28^ and as a single-photon source.^29,30^ Plasmonic properties of metal films^31−34^ and various nanostructures including metallic nanoparticles,^35−41^ nanorod arrays,^42−44^ nanoslit cavities,^45,46^ and nanohole arrays^47−52^ have been studied with the purpose of amplifying the fluorescence of QDs. However, few of the studies investigate plasmon-enhanced fluorescence for single QDs,^34,41,43,49^ because, as an example, positioning single QDs in plasmonic nanostructures in a controlled manner remains challenging. Hence, the majority of the reported studies focus on the spectral behavior of QDs in plasmonic nanostructures as one single entity including the enhanced fluorescence of QDs immobilized inside nanoholes.^47,48,50^ Nanoholes several micrometers apart from one another in relatively thick metal films, such as those in zero-mode waveguides (ZMWs),^53,54^ have also been found to enhance the photoluminescence (PL) intensity of single QDs by 2.5 times^49^ via the excitation of localized surface plasmons. Obviously, such ZMW nanoholes are isolated ones and no optical coupling exists among them. Concurrently, selective surface functionalization techniques have been well-investigated for immobilization of biomolecules onto the bottom of nanoholes in metal films.^5,55^

In this work, we present a simulation-assisted optimization of nanohole array design starting from an ultrathin Al film with the nanoholes optically coupled with one another. The objective of our study is to design coupled nanoholes with the capability of enhancing the PL of single QDs. This focus is distinctive from those earlier studies devoted to the fluorescence of QDs in coupled nanoholes yet as one single entity^47,48,50−52^ or those others centered around the emission from single QDs trapped in isolated nanoholes.^49,53,54^ The simulation was implemented on COMSOL Multiphysics and optimized by evaluating various conceivable imperfections with experimental nanoholes, including the rounded edge of nanohole rims and the inhomogeneity of nanohole size and shape. The experiment considered the importance of the nanohole period, along with the nanohole diameter and depth. A maximum 5-fold enhancement in fluorescence intensity was achieved statistically for single QDs selectively immobilized in an optimized Al nanohole array, compared with that of single QDs deposited on a bare glass substrate as reference. This study shows a promising route to nanohole-boosted fluorescence toward single-fluorophore-based biosensing.

The nanohole arrays in the Al film were fabricated using electron-beam lithography (EBL) in combination with reactive-ion etching (RIE). Since QDs usually have a wide absorption range in the blue region of visible light and higher extinction coefficients at shorter wavelengths, Al is chosen because it is well-known to possess good plasmonic properties at UV and visible wavelengths.^56^ Furthermore, the native oxide layer present on the aluminum surface helps in preventing fluorescence quenching due to the physical separation between QDs and Al. The process flow is schematically summarized in Figure 1a. First, a 30 nm thick Al film was magnetron-sputtered onto a glass substrate after standard cleaning (see *Nanohole Array Fabrication*). Next, EBL was utilized to define circular patterns in the resist layer spun-coated on the Al film. Then, RIE was used to transfer the patterns to the Al film. The fabricated nanohole arrays were characterized by means of scanning electron microscopy (SEM; see Figure 1b) and cross-sectional view with a 10° tilt (Figure 1c) of a square array of nanoholes 100 nm in diameter. The optimization of experimental Al nanohole arrays was facilitated by implementing a model on COMSOL Multiphysics to match the plasmonic peak with the excitation wavelength of the optical microscope. The RIE process was optimized to minimize overetching into the underlying glass in order to avoid sharp variations in electric field radially at the bottom of the nanohole predicted by simulation (Supporting Information Figure S2). Such variations represent the inherent inhomogeneity in electric field and would inevitably amplify the spread in fluorescence enhancement because the location of subsequently loaded QDs in the nanoholes is intrinsically random (see more below).

**Figure 1:**
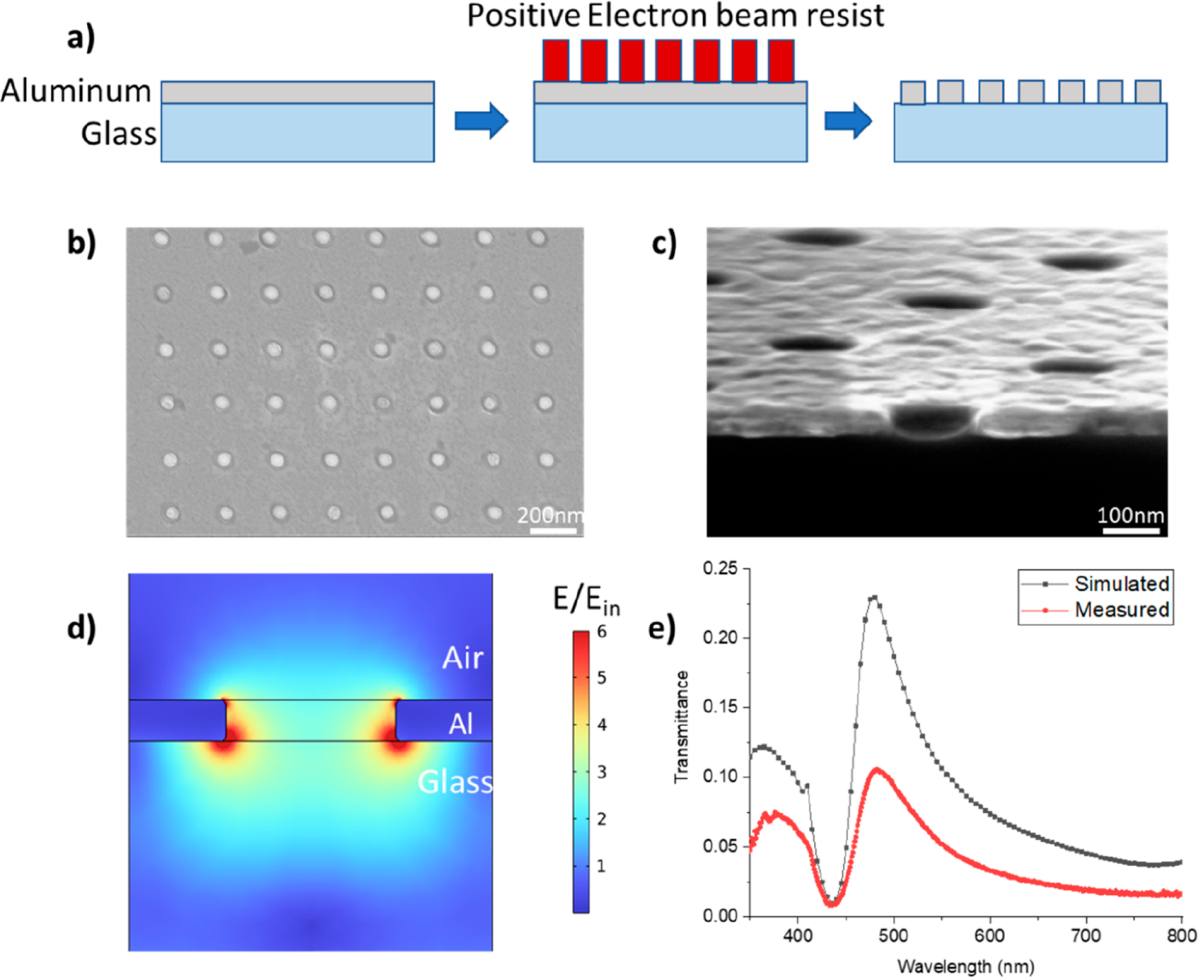
Fabrication and characterization of nanohole arrays. (a) Schematic fabrication process flow for nanohole arrays in a 30 nm thick Al film deposited on glass substrate. (b) SEM top-view image of a fabricated nanohole array on glass. (c) SEM cross-sectional view image of a nanohole array on silicon oxide coated silicon substrate. (d) Simulated electric field enhancement on the plane of the central cross-section of a nanohole with rounded edge in an array under excitation at 475 nm wavelength (transmittance peak), with *t* = 30 nm, *D* = 130 nm, and *p* = 270 nm. The color bar is in linear scale. (e) Simulated and measured transmission spectra of the nanohole array in air with identical array parameters in panel d and with light incident from the glass side (bottom).

Transmittance in nanohole arrays was utilized to calibrate the COMSOL simulation. The optimum is to have the transmittance peak (i.e., plasmonic peak) appear close to the absorption peak (about 515 nm in Figure S6). Usually, four structural parameters are critical for the optical properties of a nanohole array: thickness and specific optical properties of the metal film, diameter of nanoholes, and period of the array. The design of our nanoholes was optimized to place the primary plasmonic peak around the excitation wavelength of 515 nm and simultaneously to have the maximum electric field intensity inside the nanoholes in water environment; see Figures S5 and S6. In the simulation, the thickness of the Al film, *t*, the diameter of nanoholes, *D*, and the period of arrays, *p*, were optimized to be 30 nm, 110 nm, and 300 nm, respectively; see Figure S1d. The measured optical properties of Al were input values in the COMSOL model. To calibrate the simulation model, the transmittance spectrum of another nanohole array (different from that in Figure 1c) with *D* = 130 nm, *t* = 30 nm, and *p* = 270 nm was measured in air. The enhancement in electric field intensity for the designed nanohole compared to the electric field intensity for bare glass at the transmittance peak, i.e., 475 nm in air, is shown in two dimensions in Figure 1d. An excellent agreement between simulation and experiment is shown in Figure 1e for the transmittance with respect to the position and shape of the two peaks. The inevitable inhomogeneity of fabricated nanoholes in size and shape was found of little influence on the simulation outcome; see Figure S1. Process-induced rounding of the Al rim also had insignificant effect (Figure S2 versus Figure 1d).

The enhanced electromagnetic field by nanohole arrays is expected to directly lead to an increased PL of single QDs selectively immobilized in the nanoholes. To facilitate selective immobilization, poly(vinylphosphonic acid) (PVPA) and biotin-poly(ethylene glycol) (PEG)-silane were, respectively, used to functionalize the Al (covered by a native aluminum oxide layer) surface^5^ and the glass (silicon oxide) surface.^55^ The nanohole chip was then immersed in the solution with commercially available streptavidin-conjugated QDs (geometrical details in Figure S3) to allow for loading of the QDs into the nanoholes by natural diffusion and precipitation. It is well-established^58,59^ that silane covalently binds to silicon oxide and biotin covalently binds to streptavidin. The QDs, in the CdSe-ZnS core–shell configuration, would, then, become captured at the bottom of the nanoholes via the strong streptavidin–biotin binding, as schematically shown in Figure 2a. Few QDs could remain on the Al surface due to the PVPA passivation blocking their adsorption. The ZnS shell, polymer shell, streptavidin, and native aluminum oxide layer could keep the CdSe core from being in direct contact with Al, thereby preventing fluorescence quenching by Al. Photoluminescence measurements of single QDs both in nanohole arrays and on a reference bare glass substrate (also functionalized with silane-PEG-biotin) were carried out under the same conditions to quantitatively compare their photoluminescence intensities.

**Figure 2:**
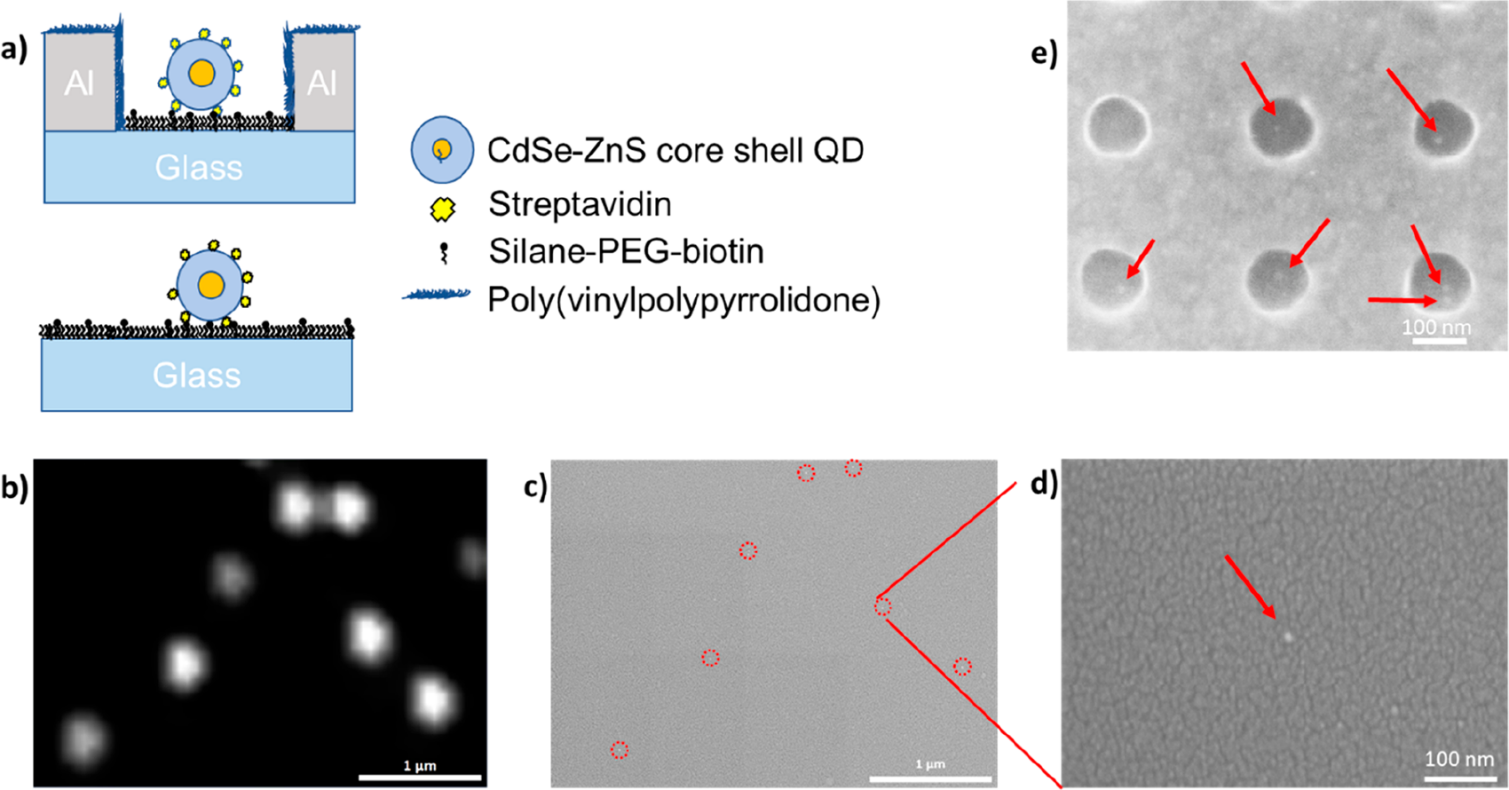
Surface functionalization for area-selective QD immobilization. (a) Schematic of a single QD immobilized in a surface-functionalized Al nanohole (up) versus that on the surface-functionalized glass (bottom) via the streptavidin–biotin conjugation. (b) PL image of QDs on bare glass substrate. (c) SEM view of the QDs in panel b. (d) Enlarged SEM view of one QD in panel c. (e) SEM image of single QDs in nanoholes. Characterizations performed in air.

The concentration and volume of the QD solution were optimized to ensure that the average distance between two adjacent immobilized QDs was larger than the diffraction limit so that the detected bright spots in fluorescence images were signals from single QDs rather than clusters. To further confirm this assertion, fluorescence images were correlated to scanning electron microscopic (SEM) images for a typical sample on a glass substrate, and an example is shown in panel b versus panel c of Figure 2. The enlarged SEM image in Figure 2d confirms the allocation of an isolated, nonclustered QD. In the case of loading QDs in a nanohole array, the filling efficiency should follow the Poisson distribution.^5^ A high yield of single QDs in the nanoholes could be achieved in some areas of the array as seen in the example in Figure 2e. However, the precise position of the QDs successfully loaded in the nanoholes varied from hole to hole, which could amplify the spread of PL intensity distribution (see below). It is worth emphasizing that in this case all detected PL signals were from the QDs inside nanoholes, because the light was shone from the glass side and so was the emission detected, while the few remaining QDs on the Al surface could not be excited due to the opaque Al film. This is one of the advantages of combining an inverted fluorescence microscope with nanohole arrays in thin metal films on glass substrate. However, the precise position of QDs in nanoholes appears to be random as seen in Figure 2e. The consequence of this randomness is a broadened enhancement factor as will be discussed below.

By recording the time traces of single QDs, the blinking characteristics of the QDs could be unveiled. Representative blinking characteristics of a single QD on a bare glass substrate in Figure 3a and that in the nanohole array in Figure 3d are shown, respectively, in Figure 3b,c. The intensity difference between the on and off state determines the PL intensity of the QDs under study; see the statistical curves to the right side in Figure 3b,e. The histogram of PL intensity for the QDs on the bare glass substrate shows a peak around 1300 in Figure 3c and that in the nanohole array with *p* = 300 nm peaks around 6300 in Figure 3f, which yield a maximum of 5-fold enhancement (i.e., 6300/1300).

**Figure 3:**
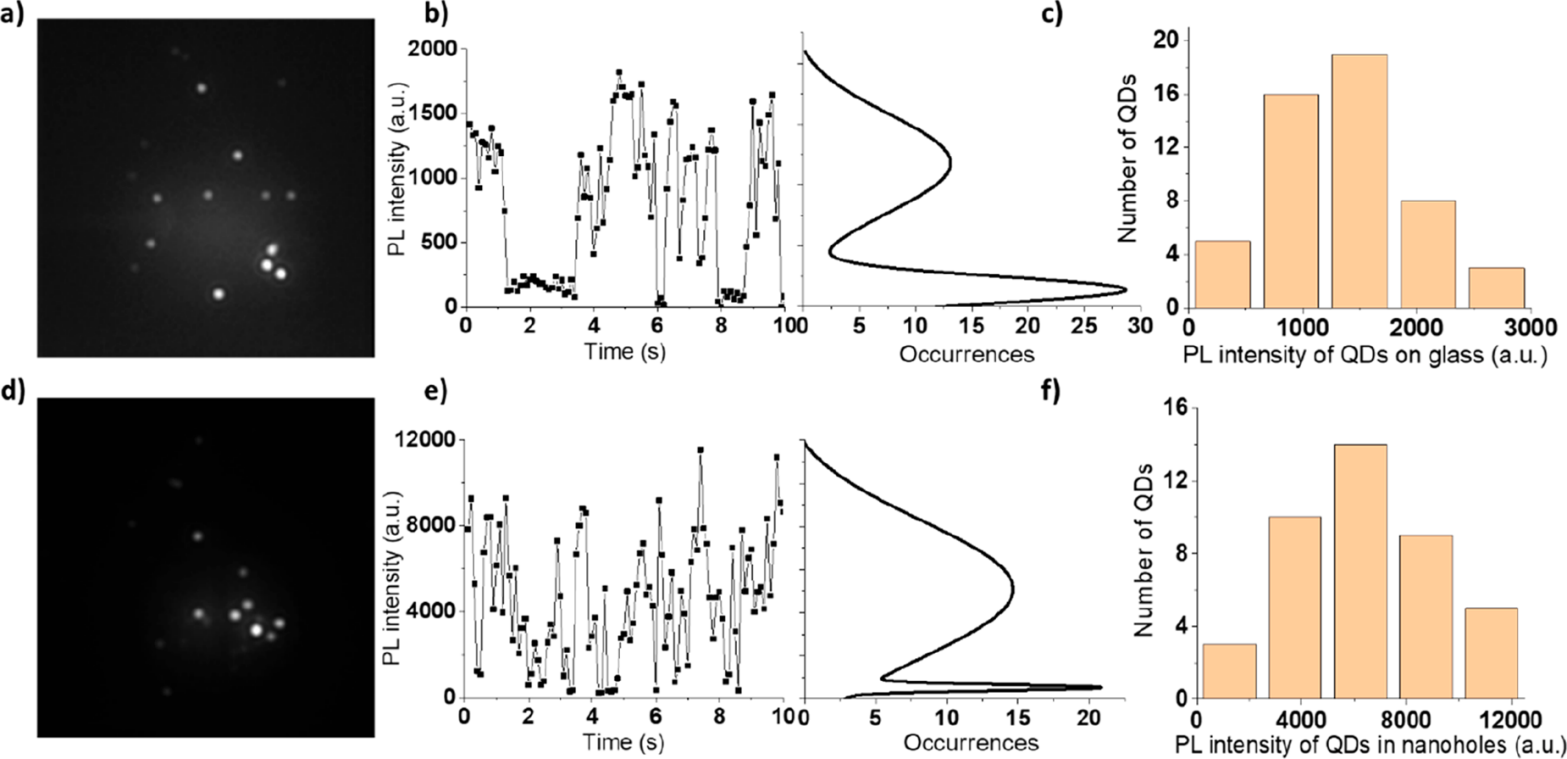
Photoluminescence of single QDs in water. (a–c) Photoluminescence image, time trace, and statistics of single QDs immobilized on glass and (d–f) corresponding information for single QDs in the nanohole array of *t* = 30 nm, *D* = 110 nm, and *p* = 300 nm. View size for both panels a and d: 25 μm × 25 μm.

Blinking is obvious for the QDs both on the bare glass substrate and in the nanoholes although; it appears differently, i.e., three large steps spanning the high on state and the close-to-zero off state in Figure 3b versus more frequent spike-like large alternations in Figure 3e. Characterization of the distribution of the on-state ratio (Figure S4) revealed that the overall duty time (i.e., fraction of time spent in the on state) of QDs did not differ much for their placement on bare glass substrate or in nanoholes. However, when the QDs in the nanoholes did blink, it came, as expected, with a much larger difference in PL intensities between the on and off states (Figure 3e). The distribution of PL intensity in Figure 3c is a consequence of the inherently inhomogeneous emitting efficiency of QDs. The inhomogeneous distribution of electric field in a nanohole (Figure S5) widens the PL intensity distribution in Figure 3f as a result of the randomness in QD location inside the nanoholes, as seen in Figure 2e.

The fluorescence enhancement factor is equal to the product of the gain in excitation intensity, quantum yield, and collection efficiency.^57^ Caution should be exercised to make sure that the fluorescence saturation regime is not reached when conducting measurements. Large locally enhanced electric field can be generated by plasmonic nanostructures, which is vital in the regime where the excitation intensity dominates. The observed PL intensity enhancement is mainly attributed to the effect of an enhanced local electromagnetic field inside the nanoholes. To further explore this effect, PL intensity of QDs immobilized in five nanohole arrays of five different periods, i.e., *p* = 240, 270, 300, 330, and 360 nm, was extracted from their time traces. The final enhancement factors are shown in Figure 4, with the summarizing diagram in the middle surrounded by the corresponding histograms. Also included in the figure is the simulation data of the average electric field enhancement factors over the whole nanohole volume. The simulated transmittance spectra and the electric field distribution of these arrays are displayed in Figure S6. The PL intensity enhancement factor is found to be sensitively dependent on *p* with the QDs inside the nanohole array of *p* = 300 nm showing the highest enhancement factor. Although numerically different, the simulation is seen to excellently reproduce this trend. For the nanohole array of *p* = 330 nm, the difference between simulation and measurement is the largest among the studied arrays. This observation can be accounted for by considering the electric field distribution in Figure S6; the highest electric field enhancement is found along the top edges of the nanoholes in the *p* = 330 nm array, while the QDs are immobilized on the bottom surface of the nanoholes, thereby leading to a lower experimental PL enhancement than the simulated counterpart. The difference in absolute values between experiment and simulation is partially attributed to the simplified model used in the simulation as QDs are mostly landing in the middle of the nanoholes rather than to the edge where the electric field is the highest. Nonetheless, it is indicative from the data in Figure 4 that the enhancement effect is to diminish when the nanoholes are further separated (such as the nanoholes in a ZMW) and become no longer optically coupled. The effect of nanopore array on quantum yield is hard to quantify due to the inhomogeneous distribution of local electromagnetic field and the random positioning of single QDs inside the nanoholes. Nonetheless, the effect on quantum yield is expected to be negligible from the simulated results of a published study on single upconversion nanoparticles in gold nanohole arrays.^60^

**Figure 4:**
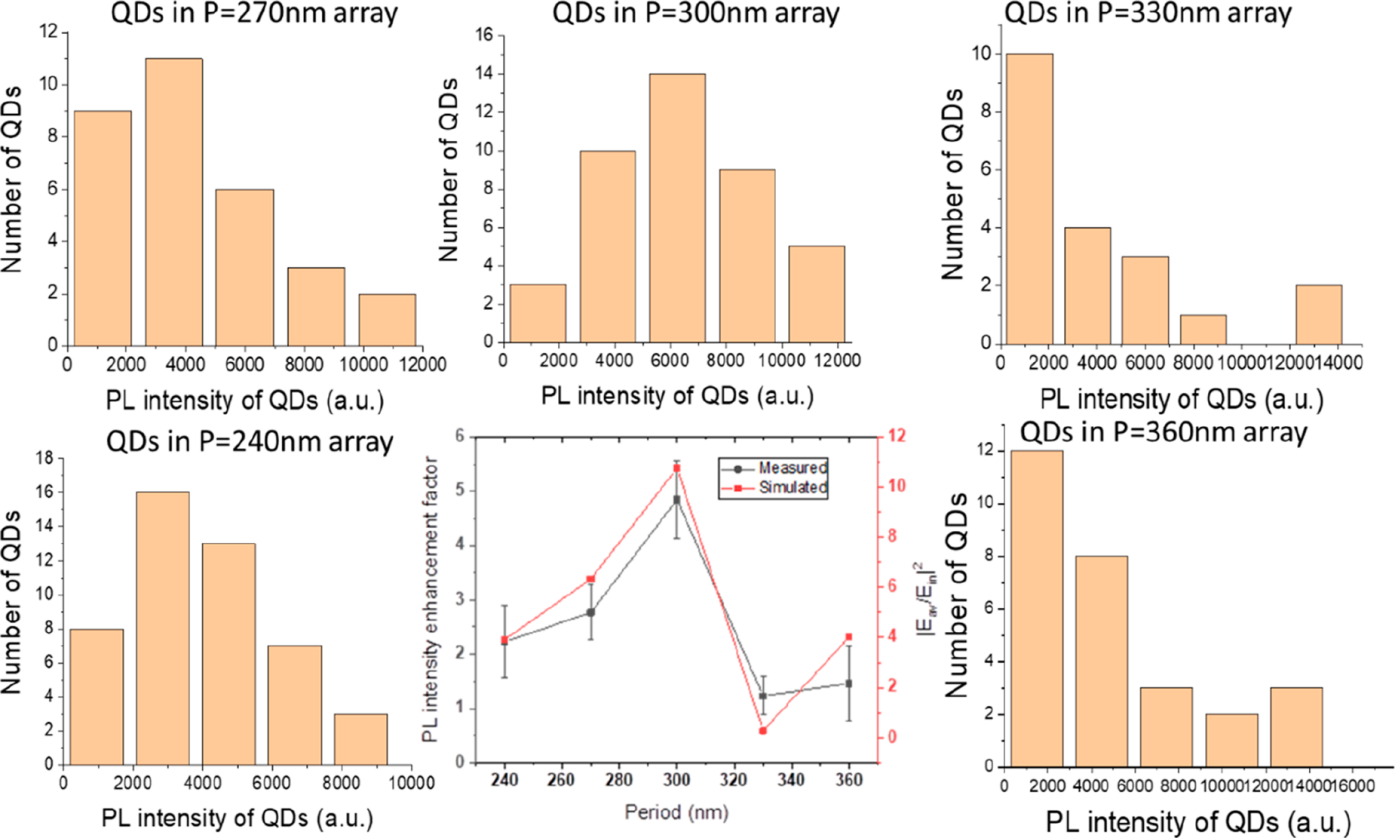
Photoluminescence in water of single QDs in nanohole arrays of different periods. Measured PL intensity enhancement factor in comparison with simulated average electric field enhancement factor in the nanohole volume for nanohole arrays of five different periods *p* = 240, 270, 300, 330, and 360 nm, with *t* = 30 nm and *D* = 110 nm. The statistical PL results for the five cases are displayed around the central figure.

As for the collection efficiency, a periodically coupled subwavelength nanohole array does not affect the angular distribution of emission from single QDs in the nanoholes, as shown from the simulated far field electric field intensity in Figure S7. In contrast, isolated nanoantenna or nanoscatters have been reported to affect the angular distribution of single fluorophores.^61,62^ Besides, the experimental optical setup was the same used for the PL measurements. Therefore, the light collection capability should remain the same. Hence, the enhanced local electric field is concluded to be predominant for the PL enhancement in this work.

A final note is made as follows. It is important that a laser source of high monochromaticity be used for excitation in the PL measurement for plasmonic study. When using a 475 nm light-emitting diode (LED) as the light source to excite similar QDs but with the 585 nm emission peak, a quenching effect was observed leading to a 5-fold reduction in PL intensity for the QDs in the nanohole array with the plasmonic peak around 475 nm instead, both in water and air (Figure S8). The root cause for the quenching effect is that, with the wavelength ranging from 450 to 488 nm, the LED light is not so monochromatic as a laser source. The shorter wavelengths around 450 nm at which the QDs exhibit a high extinction coefficient are strongly reflected by the Al structure with nanohole arrays.

In conclusion, this study shows a simulation-experiment tandem design and optimization promising for single-fluorophore-based studies that require strong fluorescence signals for high sensitivity in biosensing. With the assistance of numerical simulation, the structure of nanohole arrays in aluminum films to match plasmonic peak with the excitation wavelength was designed in order to reach the maximum enhancement of local electromagnetic field, thereby enhancing PL intensity from single QDs in the coupled nanoholes. The optimized design was experimentally realized based on standard silicon process technology, demonstrating an excellent agreement between simulation and experiment in several aspects, including transmittance and PL intensity enhancement. The maximum factor of PL intensity enhancement achieved was 5 for single QDs loaded in coupled nanoholes, in comparison to the PL intensity of QDs deposited on a bare glass substrate. The PL enhancement was experimentally verified to mainly be due to the raised local electromagnetic field, by varying the period of arrayed nanopores.

*Nanohole Array Fabrication*.The nanohole arrays in Al films were fabricated by combining electron beam lithography (EBL) with plasma-based dry-etching. A 170 μm thick borosilicate glass substrate (coverslip) was first cleaned by following standard protocols: immersed in the solution with a mixture of ammonia, hydrogen peroxide, and deionized water (NH_4_OH:H_2_O_2_:H_2_O) at 60 °C for 10 min. After rinsing in deionized water for 1 min, the coverslip was immersed in the solution with a mixture of hydrochloric acid, hydrogen peroxide, and deionized water (HCl:H_2_O:H_2_O_2_) at 60 °C for 10 min. After drying with nitrogen, an oxygen/nitrogen plasma (Tepla 300) treatment was used to further remove organic residues from the coverslip surface. Next, an Al film about 30 nm in thickness was sputter-deposited on the cleaned coverslip in a magnetron sputter (Von Ardenne CS730S) at power 500 W for 15 s. Then, a 200 nm thick AR-P 6200.09 positive electron-beam resist (Allresist GmbH) was spun atop the coverslip followed by soft bake on a hot plate at 150 °C for 1 min. Circular patterns were written on an EBL system (nB5, NanoBeam, U.K.) with 1 nA current and 80 kV accelerating voltage. After development in AR600-546 developer (Allresist GmbH) for 2 min and rinsing in deionized water for 1 min, a postbake was carried out at 130 °C for 1 min. Subsequently, the sample was etched in an inductively coupled plasma-reactive ion etching (ICP-RIE) system (PlasmaTherm SLR) for 25 s, with 30 sccm (standard cubic centimeter per minute) Cl_2_ and 50 sccm BCl_3_ gas flow, 6.0 mTorr pressure, and 80 W bias power. Thereafter, flowing a mixed gas of SF6 and O_2_ removed residue chloride. After etching, the residue resist was removed using remover AR600-71. Finally, the chip (on the coverslip, glass substrate) was rinsed with deionized water for 1 min and blown dry with nitrogen gas.

*Surface Functionalization and Quantum Dot Immobilization*. The Al surface (with native aluminum oxide) was functionalized with PVPA (Polysciences) by immersing the chip into 2% PVPA solution at 80 °C for 2 min. After dipping in deionized water, nitrogen blown dry, and baking at 80 °C for 15 min, the bottom in the Al nanoholes, i.e., the surface of the glass in the nanoholes, was functionalized with biotin-PEG-silane, after immersion in 100 μM biotin-PEG-silane and mPEG-silane (Laysan Bio Inc.) in an anhydrous toluene solution with 10 mM glacial acetic acid for 24 h. Then, a sticky silicone 2 × 2 wells (Ibidi) was used as a reservoir. 100 μL of 1 pM streptavidin-conjugated CdSe-ZnS core–shell QDs, each QD having 5–10 streptavidin molecules and with a diameter of 16–17 nm in total and emission peak around 655 nm (Invitrogen of Thermo Fisher Scientific) in Tris-buffered saline (1× TBS, pH 8.0, Alfa Aesar) with 10% BSA (Thermo Scientific), was added into the wells and incubated for 2 h. Subsequently, a repeated strong wash with 1× TBS for three times was carried out to remove unbound QDs. The streptavidin on the surface of QDs covalently binds with the introduced biotin molecules on the surface of glass.

*Structural and Optical Characterization*. Both the fabricated nanohole arrays and dispersed single QDs on glass were characterized by means of SEM (LEO 1530, Zeiss) at 10 kV accelerating voltage. For the latter sample, a metallic bilayer of about 3 nm thick Au/Pd was first sputter-deposited to reduce charging effect.

An inverted wide-field fluorescence microscope (Colibri 5, Zeiss) with 475 nm LED light source and a Hamamatsu CCD camera (Orca Flash 4) were used to correlate the fluorescence images of QDs on glass to their SEM images. A 100×/1.3 NA oil-immersion objective was used to both illuminate the sample and collect fluorescence emission from the sample.

A microphotoluminescence (μPL) setup, the core system of which is an inverted microscope (Zeiss Axio Observer Z1), was used to measure the PL intensity of single QDs both on glass and in Al nanoholes. A 515 nm laser with 5 mW output power was used to excite QDs, and a 63×/0.75 NA objective with glass-thickness correction was used to focus the laser beam and to also collect PL signals. A 650 nm bandpass filter (Coherent 35-5107-000) and a CCD camera were used to record PL time traces (0.1 s/frame, 10 s) of single QDs.

Transmission measurement was carried out on an ultraviolet–visible–near-infrared spectrophotometer (Lamda 900, PerkinElmer). An integrating sphere was used during the measurement to obtain the total transmission of the Al nanohole arrays.

*PL Intensity Analysis*. All time traces of single QDs were analyzed using the ImageJ software. First, a picture showing an average intensity of the total 100 frames was retrieved. Next, the maximum number of pixels of each single QD was found. Then, 5 × 5 pixels surrounding the maximum pixels were selected as the signal plus background, while the neighboring 5 × 5 pixels were chosen as background. Subtracting the latter from the former yielded the PL signals of single QDs. The inherent inhomogeneous intensity distribution of the laser spot was also calibrated for the PL intensity of single QDs. By plotting the time traces of the integrated intensity of these pixels, the blinking properties of QDs could be unveiled. And the difference in PL intensity between the on and off state was calculated as the PL intensity of QDs.

*Simulation*. The finite element method based commercial software COMSOL Multiphysics was used to simulate the optical properties of the nanohole arrays in Al films with the wave optics module. One-quarter of a unit cell of the nanohole arrays with perfect electric conductor (PEC) and perfect magnetic conductor (PMC) boundary conditions was built up to represent a two-dimensional infinite array. A linearly polarized plane wave was set to incident from the glass side. Perfect matched layers (PML) were used to absorb waves. A fillet of 5 nm radius was introduced to control the rounding radius of the nanohole edges, and the optical properties of Al film were interpolated using the measured values. Fine physics-controlled mesh was used. Transmission spectra, electric field distribution, and average electric field enhancement factor over the nanohole volume were calculated with the built-up model. The background electric field amplitude of incident linearly polarized plane wave is set to be 1 V/m. The average electric field enhancement factor over the nanohole volume is then equal to the average electric field values over the nanohole volume. Far-field electric field intensity can be plotted by putting an electric point dipole inside the nanohole with the dipole moment parallel with the incident electric field.

## Supporting Information Available

Additional simulation results, materials, and PL measurement results (PDF)
