# Supplementary material for: Plasmon-Enhanced Fluorescence of Single Quantum Dots Immobilized in Optically Coupled Aluminum Nanoholes
Source: J Phys Chem Lett. 2023 Feb 27;14(9):2339–46. doi: 10.1021/acs.jpclett.3c00468 (PMC10009806; doi:10.1021/acs.jpclett.3c00468)
Supplement: Supplementary file 1 — jz3c00468_si_001.pdf [file jz3c00468_si_001.pdf]

Supporting Information for

Plasmon-Enhanced Fluorescence of Single Quantum Dots Immobilized in Optically Coupled Aluminum Nanoholes

Yupeng Yang[□], Apurba Dev[□], Ilya Sychugov[†], Carl Hägglund[§], and Shi-Li Zhang[□]*

[□]Division of Solid-State Electronics, Department of Electrical Engineering, The Ångström Laboratory, Uppsala University, SE-751 03 Uppsala, Sweden

[†]Division of Photonics, Department of Applied Physics, School of Engineering Sciences, KTH Royal Institute of Technology, SE-100 44 Stockholm, Sweden

[§]Division of Solar Cell Technology, Department of Materials Science and Engineering, The Ångström Laboratory, Uppsala University, SE-751 03 Uppsala, Sweden

[*]Email: shili.zhang@angstrom.uu.se

Number of pages: 7 Number of tables: 0 Number of graphs: 8

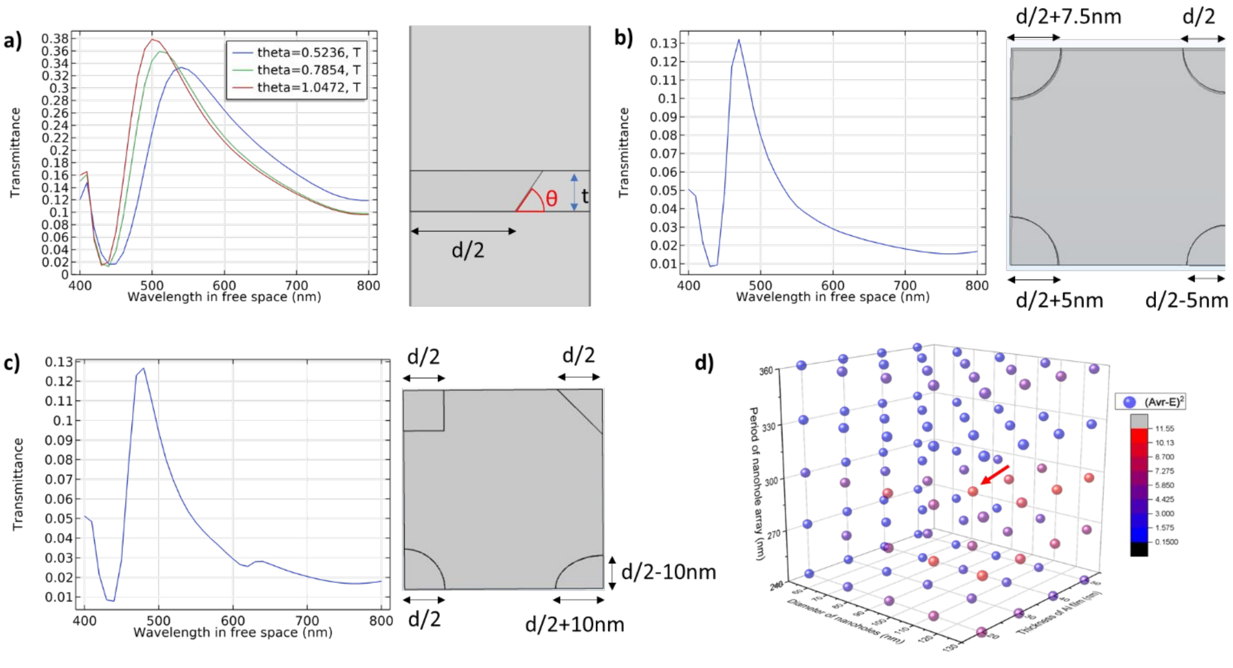

Figure S1. Simulated effect of structural parameters on transmittance spectrum (a-c) to be compared with the data in Figure 1e. (a) Effect of the slope of nanohole sidewall with 30°, 45°, and 60° tilt angles. A unit cell is used to represent extreme cases of the effect of (b) inhomogeneous size and (c) shape of nanoholes on transmittance spectrum. (d) Simulated square of average electric field enhancement factor over the nanohole volume with all combinations of three structural parameters: period of nanohole array, diameter of nanoholes, and thickness of the aluminum film under excitation at 515 nm. The bubble with the highest value of enhancement factor is indicated by a red arrow.

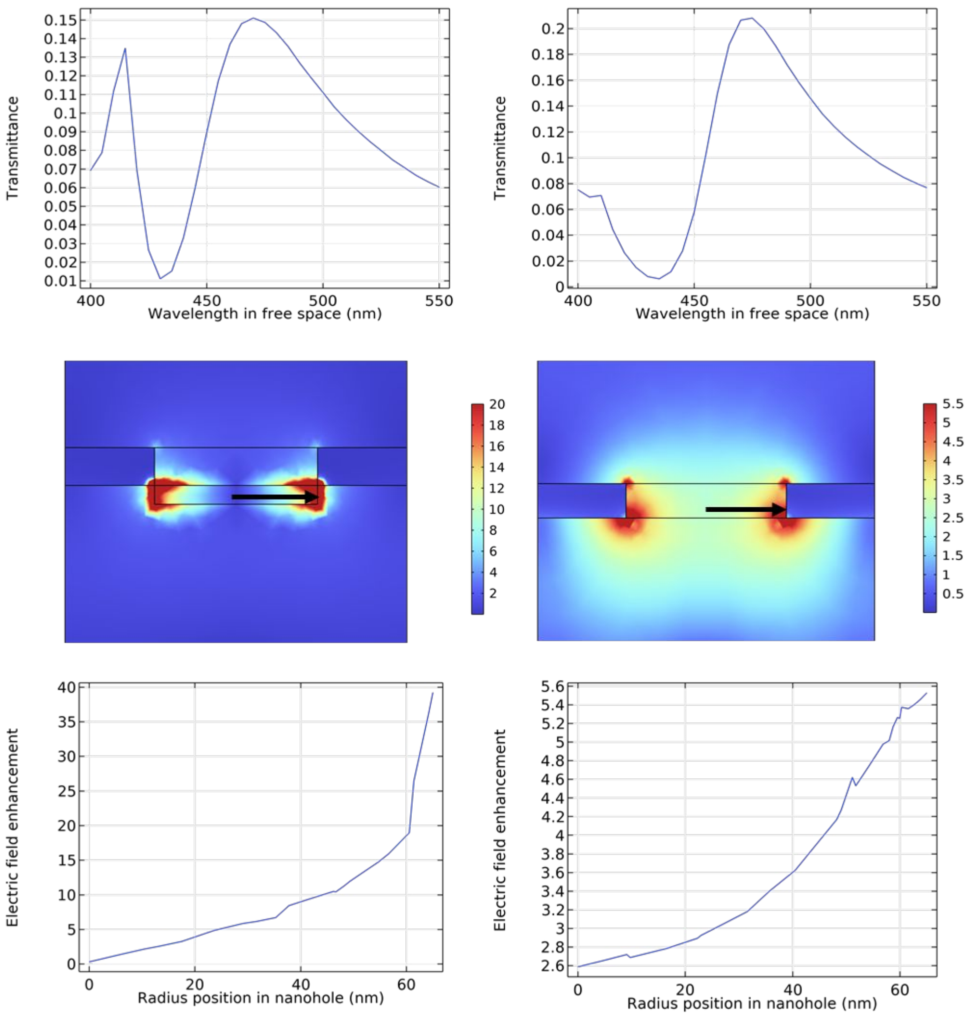

Figure S2. Effect of over-etch into glass (left column) with reference to ideal non-over-etch (right column) on transmission (upper row), electric field distribution contour (middle row), and radial distribution of electric field along the arrow at 8 nm distance height from glass surface (d=130 nm, t=30 nm, p=270 nm). In both cases, the edges of nanoholes are sharp, not rounded.

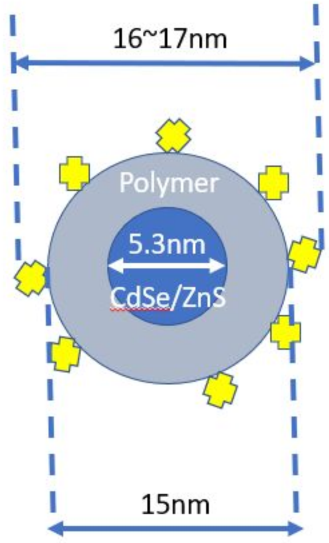

Figure S3. Schematic of size details of the commercial QDs. 5~10 streptavidin molecules are conjugated on the surface per QD.

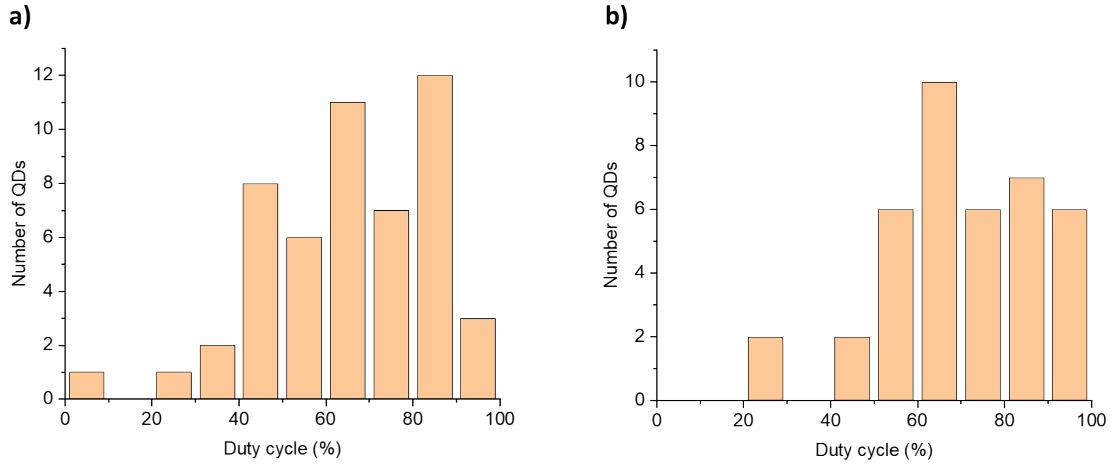

Figure S4. Distribution of duty cycle (fraction of time spent in ON-state) of QDs (a) on glass and (b) in nanohole array with 300 nm period.

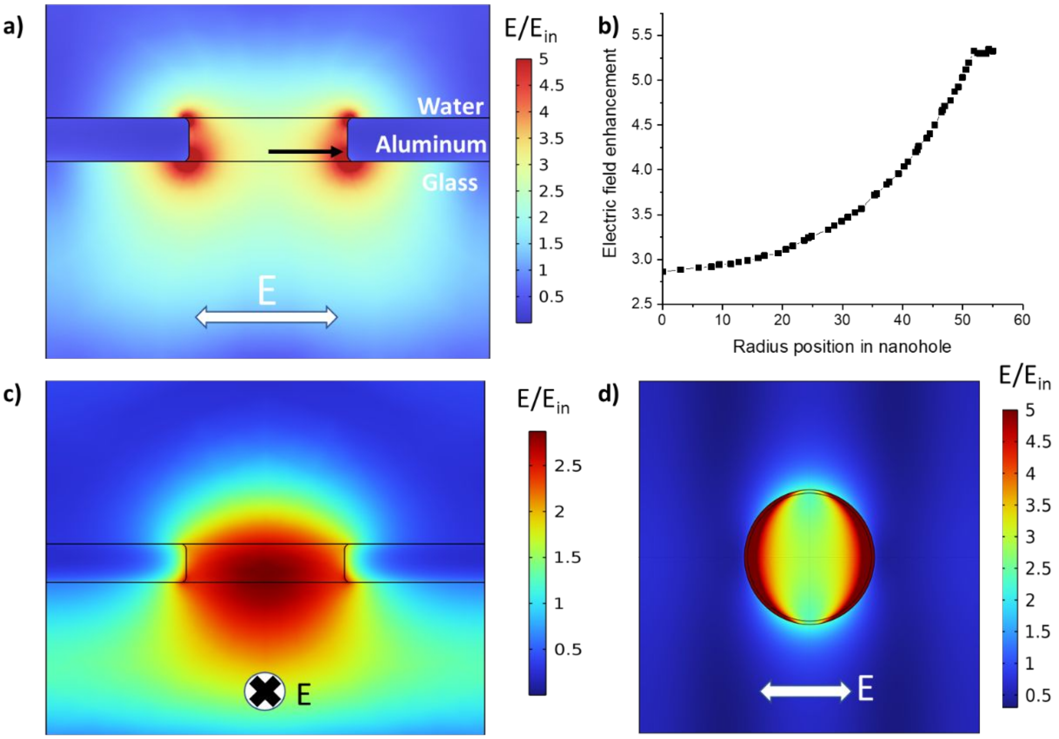

Figure S5. Simulated distribution of electric field in the nanohole region in water (*d*=110 nm, *p*=300 nm, *t*=30 nm). (a) Cross section parallel to the polarization of incident electric field. (b) Extracted numerical data along the arrow in (a), which is 8 nm above the glass surface in the nanohole and starts from the nanohole center till the edge. (c) Cross section perpendicular to the polarization of the incident electric field. (d) Top view.

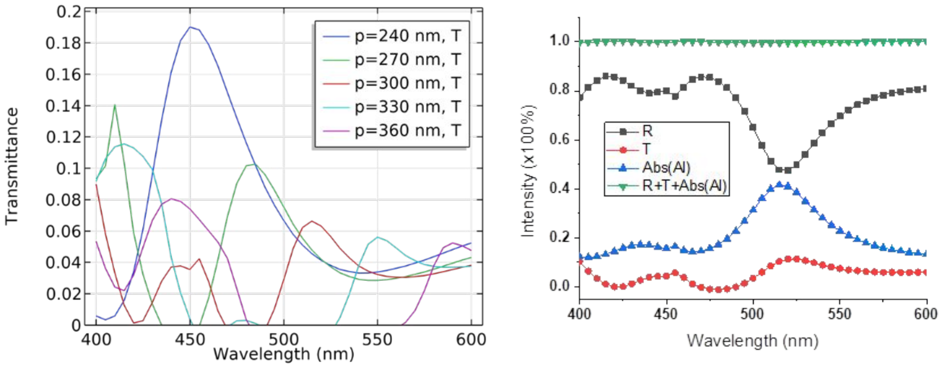

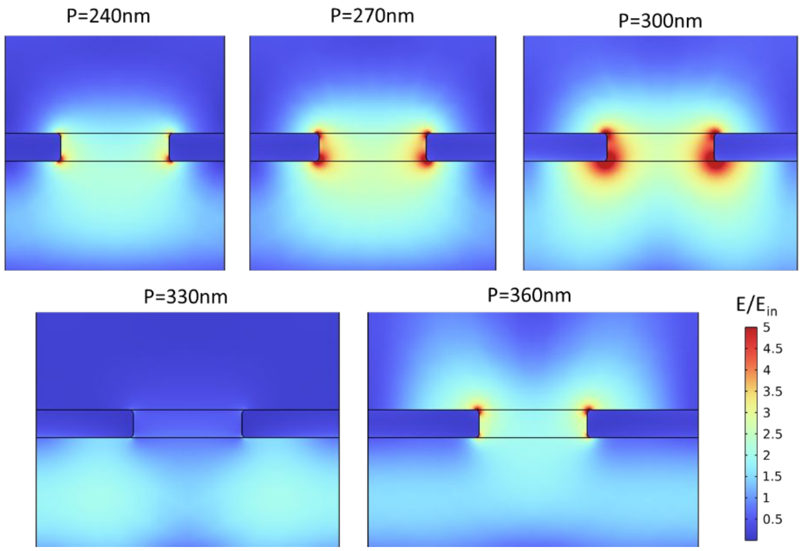

Figure S6. Simulated transmittance spectra of nanohole arrays in air with different periods while thickness is set to be 30 nm and diameter of nanoholes are 110 nm. The transmission peak redshifts from 450 nm to 590 nm when the period of a nanohole array increases from 240 nm to 360 nm as seen in the upper left figure. Reflectance, transmittance, absorptance of several Al nanohole arrays with period 110 nm and the sum of them are plotted in the upper right figure. Figures at bottom show the simulated distribution of electric field near the nanohole region of arrays parallel to the polarization of the incident electric field for different periods under 515 nm light excitation.

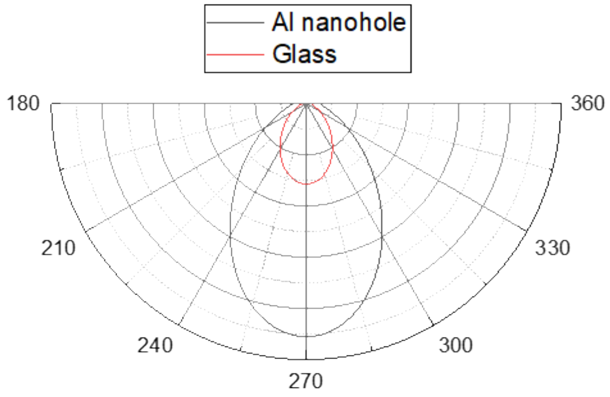

Figure S7. Simulated far field angular distribution of electric field on the glass side emitted from an electric point dipole representing a single QD located at the center of a nanohole but elevated by 8 nm from the bottom, of which the dipole moment is parallel with the incident electric field at 515 nm.

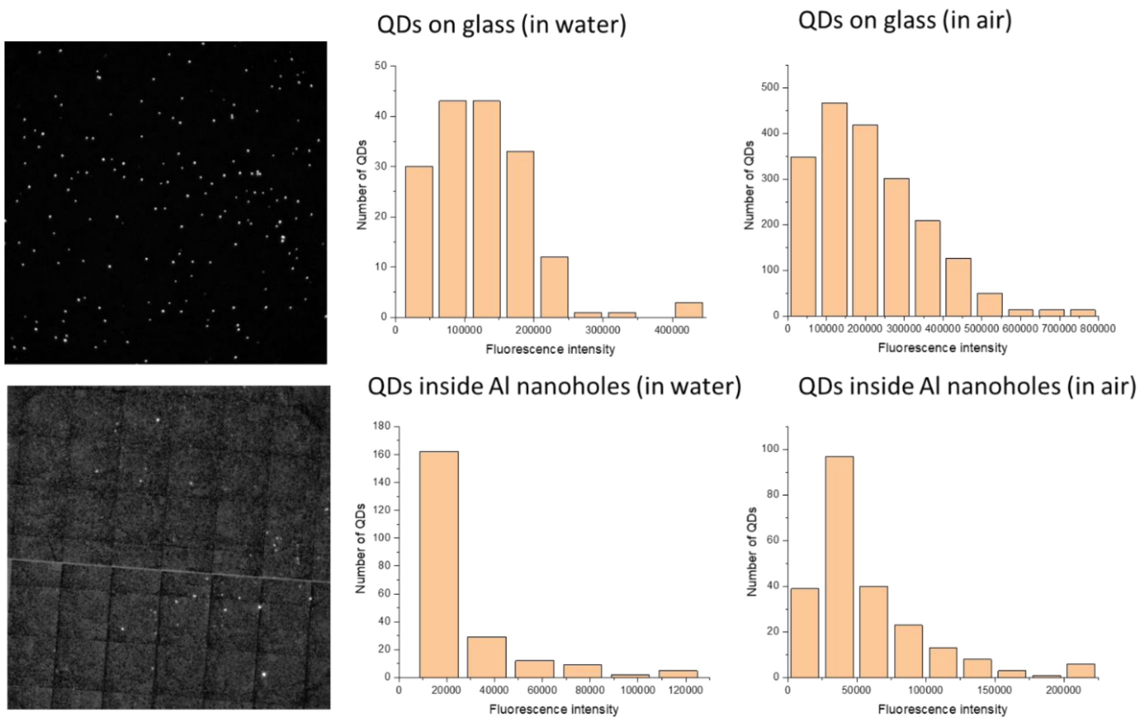

Figure S8. PL images and statistics of single QDs on glass (first row) and in nanoholes (second row) with 475 nm (±15 nm) LED excitation. View size for both images: 133 µm x 133 µm.
